# Preoperative Albumin–Bilirubin Grade With Prognostic Nutritional Index Predicts the Outcome of Patients With Early-Stage Hepatocellular Carcinoma After Percutaneous Radiofrequency Ablation

**DOI:** 10.3389/fmed.2020.584871

**Published:** 2020-11-10

**Authors:** Jingying Pan, Shuochun Chen, Guo Tian, Tianan Jiang

**Affiliations:** Department of Ultrasound, The First Affiliated Hospital of Zhejiang University, Hangzhou, China

**Keywords:** hepatocellular carcinoma, radiofrequency ablation, prognostic nutritional index, albumin-bilirubin, overall survival

## Abstract

**Background:** Prognostic nutritional index (PNI) that was designed to assess the nutritional and immunological status of patients and albumin–bilirubin (ALBI) grades can be used as an assessment tool for hepatic function. Both nutritional and immunological statuses have been reported to be independent prognostic factors of patients with hepatocellular carcinoma (HCC). This study aimed to investigate whether PNI together with ALBI could be a better predictor in patients with early-stage HCC undergoing radiofrequency ablation (RFA).

**Method:** The information of 110 patients with newly diagnosed HCC within the Milan criteria receiving RFA as the initial therapy between 2014 and 2015 was retrospectively collected. Pretreatment PNI, ALBI, and PNI-ALBI grades were calculated. Overall survival (OS) and recurrence-free survival (RFS) were estimated by the Kaplan–Meier method, and multivariate analysis was used to identify prognostic factors.

**Result:** The 1-, 3-, and 5-years OS rates of patients were 80.0, 30.9, and 23.9%, respectively. Multivariate analysis showed that the tumor size [hazard ratio (HR) = 1.966, 95% confidence interval (CI) = 1.091–3.545, *P* = 0.025], PNI grade (H = 2.558, 95% CI = 1.289–5.078, *P* = 0.007), and PNI-ALBI grade (HR = 3.876, 95% CI = 1.729–8.690, *P* = 0.001) were independent risk factors for OS, whereas only the elevated α-fetoprotein (HR = 1.732, 95% CI = 1.003–2.991, *P* = 0.049) and the size of the tumor (HR = 1.640, 95% CI = 1.015–2.647, *P* = 0.43) were independent predictors for better RFS.

**Conclusion:** This study demonstrates that preoperative PNI-ALBI grade is a simple and useful predictor for OS in patients with early-stage HCC after RFA.

## Introduction

Liver cancer is the sixth most frequent malignancy, ranked as the fifth most common in men, and ninth in women, respectively. It is the fourth leading cause of cancer-related deaths worldwide, next to lung, colorectal, and stomach cancers (1). Hepatocellular carcinoma (HCC) accounts for ~75% of all liver cancers and is the most common primary liver tumor (2).

The main risk factors for HCC include chronic infection with hepatitis B virus (HBV) or hepatitis C virus (HCV), aflatoxin exposure, etc. However, in most cases, particularly in high-risk areas, HCC develops as a sequela to protracted chronic infection with the HBV or HCV, with or without the development of liver cirrhosis (3).

Although there is a significant improvement in the clinical diagnosis and treatment of HCC, however, with the deterioration in the liver function, high recurrence rates, and distant metastasis, the rates of morbidity and mortality of HCC continue to increase (4).

Percutaneous radiofrequency ablation (RFA) is recognized as an important alternative treatment for small HCCs and cases where resection cannot be performed, due to location, tumor size, multimodality, or inadequate function. RFA can cause necrosis of the tissues of hepatic carcinoma by thermal coagulation (5). It was confirmed to be an effective therapy in patients with hepatic cirrhosis and in single HCC ≤ 5 cm in diameter or up to three HCCs each 3 cm or smaller, and there was no significant difference between the survival rate of RFA and surgical resection (6, 7). Thus, the good candidates for RFA are patients with early-stage HCC (single tumor ≤ 5 cm in diameter, or tumor number ≤ 3 with the maximum diameter of each ≤ 3 cm) (8). But if the HCC diameter is larger than 4 cm, it is not considered to be much effective (9). In RFA, a solitary inserted electrode can cause necrosis of an area with a diameter of ≤ 3 cm and ablate 2-cm tumor completely (10). Also, RFA has effects that are similar to the microwave ablation and cryoablation, but it has become more common because of the prevalence and convenience of the device. However, the methods for assessing the survival outcomes of the postoperative patients were limited.

The assessment of liver function and failure is vital in predicting overall survival (OS) of the patients with HCC. The Child–Pugh (C-P) grade has been widely used in the assessment of preoperative liver function in clinical practice, and it is based on a score derived from five parameters, including conventional liver function tests, extent of ascites, and degree of hepatic encephalopathy. However, the grading of ascites and encephalopathy can be highly subjective, which could introduce confounding evaluation (11, 12). A new model named the albumin–bilirubin (ALBI) grade was first defined by Johnson et al. (13), and it is one of the best indicators of liver function and showed better discriminative performance than C-P grade. A recent study found that the ALBI grade predicted recurrence-free survival (RFS) and OS more accurately than the CP grade in patients with HCC undergoing liver resection with curative intent (14, 15). ALBI was calculated by two objective variables (albumin and bilirubin), and it was used to stratify patients with HCC into three categories of liver function risk. The higher the ALBI grade, the poorer was the patient's outcome. For the ALBI grade, the following formula was used: ALBI = (log_10_ bilirubin (μmol/L) × 0.66) + (albumin (g/L) × −0.085) (13). ALBI values had three grades. The cutoff points were as follows: grade 0 (< −2.60), grade 1 (> −2.60 to ≤ −1.39), and grade 2 (> −1.39).

The prognostic nutritional index (PNI) was originally proposed to assess the nutritional status and predict the surgical risk in gastrointestinal surgery patients by Buzby et al. (16), and Onodera et al. corroborated this in 1984 (17). Since then, further function of PNI was investigated, and a large amount of recent studies found this index to be associated with the prognosis of patients with different types of solid tumors, such as lung cancer, gastric cancer, colorectal cancer, and esophageal carcinoma (18–21). In patients with early-stage HCC, increasing evidence shows that PNI is an effective independent factor of the OS after RFA (15). The following formula was used for PNI: serum albumin (g/L) + 0.005 × absolute lymphocyte count (per mm^3^) (17). However, the combination of ALBI and PNI, which can evaluate both the nutritional immunological status and hepatic function, has never been applied in the prognosis of the HCC patients. In this study, a new index, the PNI-ALBI grade, was put up, and its prognostic significance in HCC patients was assessed.

## Patients and Methods

The study was conducted in patients with HCC receiving RFA as initial therapy in the First Affiliated Hospital of Zhejiang University between January 2014 and December 2015.

**Figure 1:**
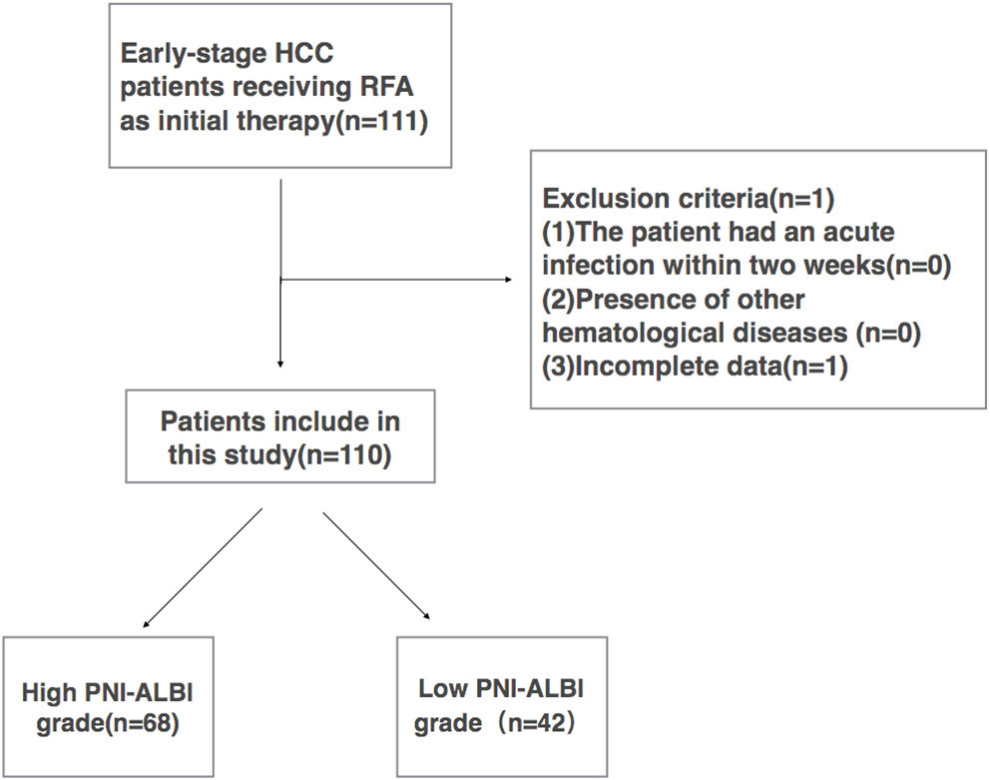
Flowchart of this study. RFA, radiofrequency ablation.

All early-stage HCC patients during the same period who met the following criteria were included in this retrospective study: (1) early-stage HCC (single tumor ≤ 5 cm in diameter, or tumor number ≤ 3, a maximum diameter of each ≤ 3 cm), (2) no extrahepatic metastasis or major vascular invasion, (3) platelet count >50,000/mm^3^, (4) patients who refused surgical treatment, (5) the patients with a pathological diagnosis of HCC, and (6) patients who had not undergone chemotherapy or other preoperative antitumor treatment before. The patients were excluded if (1) the patient had an acute infection within 2 weeks, (2) presence of other hematological diseases, or (3) incomplete data. A total of 111 patients met the inclusion criteria, and 1 patient was excluded because of incomplete data. Finally, 110 patients were enrolled in this study (Figure 1).

The subsequent clinical parameters recorded were gender; age at diagnosis; size of the tumor; conditions such as diabetes, hypertension, ascites, schistosomiasis, and cirrhosis; and HBV-DNA levels. Besides, the laboratory blood investigations were carried out before RFA, including white blood cell (WBC) count, platelet count, prothrombin time, absolute lymphocyte count, serum albumin, total bilirubin, and serum α-fetoprotein (AFP).

Authors have access to information that could identify individual participants during or after data collection. This study was provided by the Research Ethics Committee of the First Affiliated Hospital of Zhejiang University. Written informed consent was obtained from patients before treatment. The study was conducted in accordance with the ethical principles stated in the Declaration of Helsinki. All methods were performed in accordance with the relevant guidelines and regulations.

### Statistical Analysis

All statistical analyses were performed using SPSS 21.0 (SPSS Company, Chicago, IL, USA) for Windows. Categorical variables were examined using Fisher exact test. All continuous variables were expressed as the mean ± standard deviation, and the comparison between them was analyzed using the *t*-test. Spearman correlation was determined to analyze the correlation between PNI and PNI-ALBI grade. A time-dependent receiver operating characteristic (ROC) curve analysis was used to determine the cutoff values of PNI. In order to assess the ability of different models in predicting postoperative prognosis, our analysis was performed using the c-statistic equivalent to the area under the ROC curve (AUC). The RFS and OS were determined using the Kaplan–Meier method, and comparisons were determined using the log-rank test. The independent risk factors for the RFS and OS were identified using Cox regression analysis. The variables found to be significant (*P* < 0.05) in the univariate analysis were included in the multivariate analysis. A *P* < 0.05 was considered statistically significant.

## Results

A total of 110 patients were included in this study. Among them, there were 18 (16.4%) females and 92 (83.6%) males. The average age was 57.38 ±10.10 years. Sixteen (14.5%) patients had similar cases in their families. According to tumor–node–metastasis (TNM) staging system, 112 of the cases were stage I, 8 were stage II, and none of them were stage III or IV. There were 21 patients (19.1%) who had hypertension, and 19 (17.3%) had diabetes mellitus. There were 14 (12.7%) patients with ascites and 29 with schistosomiasis. A total of 80 (72.7%) patients had cirrhosis, and 101 patients had a positive HBV-DNA load. The average tumor size was 2.25 ± 0.67 cm. The size of the tumor was between 0.8 and 4.1 cm. Only one patient had HCC ≤ 1 cm in diameter, and the nodule was 0.8 cm in diameter. There was one patient with HCC ≥4 cm, and the nodule was 4.1 cm. WBC, platelet count, prothrombin time, total bilirubin, and AFP of patients before RFA are shown in Table 1.

**Table 1:** Baseline characteristics of the patients.

**Clinicopathological**	**Number (%) of patients or**
**feature**	**Mean ± *SD***
**Age**	
<55 years	43 (39.1%)
≥55 years	67 (60.9%)
**Gender**	
Female	18 (16.4%)
Male	92 (83.6%)
**Family cases**	
Yes	16 (14.5%)
No	96 (85.5%)
**Tumor size**	
>2.5 cm	30 (27.3%)
≤ 2.5 cm	80 (72.7%)
**TNM stage**	
I	112 (93.3%)
II	8 (6.7%)
**Hypertension**	
Yes	21 (19.1%)
No	89 (80.9%)
**Diabetes mellitus**	
Yes	19 (17.3%)
No	91 (82.7%)
**Ascites**	
Yes	14 (12.7%)
No	96 (87.3%)
**Splenomegaly**	
Yes	79 (71.8%)
No	31 (27.3%)
**Cirrhosis**	
Yes	80 (72.7%)
No	30 (27.3%)
**HBsAg**	
Present	101 (91.8%)
Absent	9 (8.2%)
**AFP (μg/L)**	
Normal	90 (81.8%)
Abnormal	20 (18.2%)
**ALBI grade**	
1	54 (49.1%)
2	54 (49.1%)
3	2 (1.8%)
**PNI**	
>47.2	47 (42.7%)
≤ 47.2	63 (57.3%)
**PNI-ALBI grade**	
0	42 (38.2%)
1	68 (61.8%)
WBC (×10^3^/μL)	4.39 ± 1.72
Platelet count (×10^9^/L)	106.31 ± 57.46
Prothrombin time (s)	16.02 ± 34.78
Albumin (g/L)	39.06 ± 5.16
Total bilirubin (μmol/L)	19.01 ± 9.48

*SD, standard deviation; TNM, tumor–node–metastasis; HBsAg, positive hepatitis B surface antigen; AFP, α-fetoprotein; ALBI, albumin–bilirubin; PNI, prognostic nutritional index; WBC, white blood cell count*.

**Figure 2:**
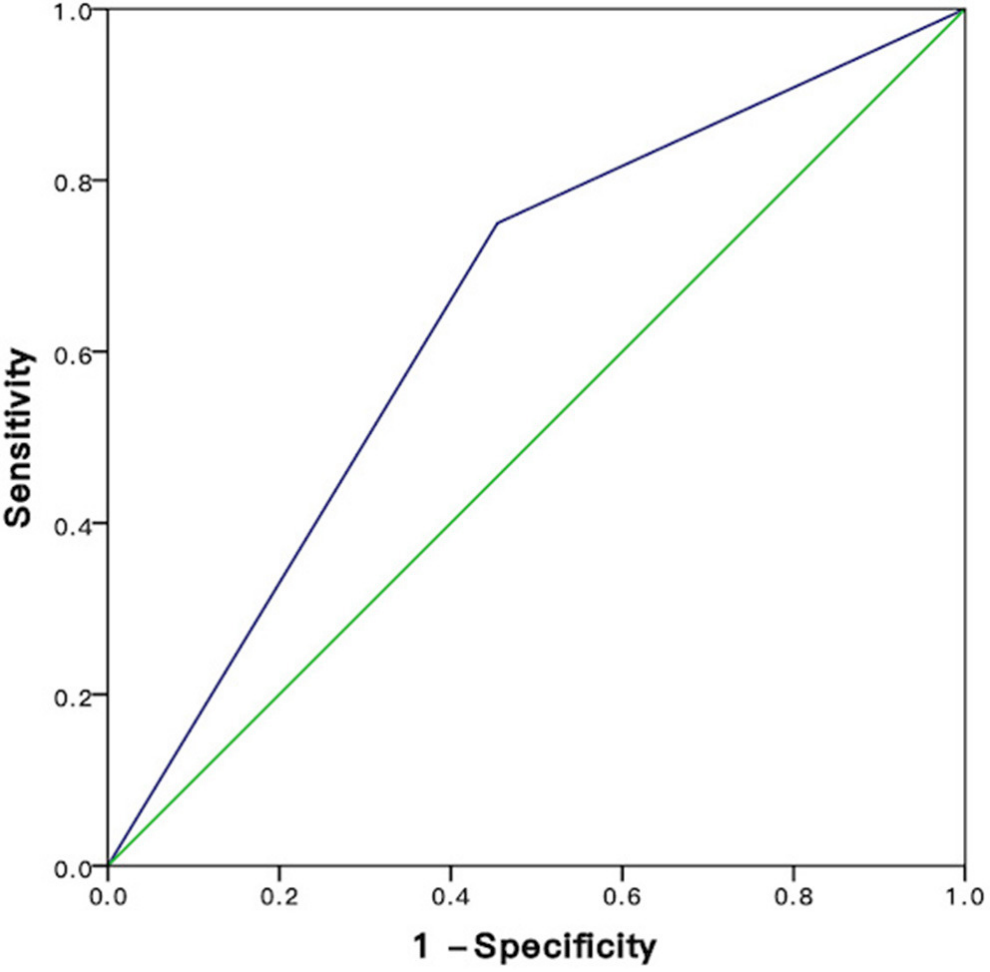
ROC analysis for sensitivity and specificity of PNI. HCC, hepatocellular carcinoma; RFA, radiofrequency ablation; PNI, prognostic nutritional index; ALBI, albumin-bilirubin.

Based on ROC curves, the cutoff value of PNI was 47.2 (Figure 2). A PNI ≥47.2 was considered as a high PNI, and a PNI <47.2 was considered as a low PNI.

Patients with a high PNI were allocated a score of 0; otherwise, the patients were allocated a score of 1. Patients with ALBI grade 0 were allocated a score of 0, grade 1 a score of 1, and grade 2 were allocated a score of 2.

The combination of the ALBI and PNI (PNI-ALBI) scores was the summation of the two scores. They ranged from 0 to 3. There were 47 patients with high PNI. The number of patients in ALBI grades 1 and 2 was 54 each. There was only one patient in ALBI grade 3. The PNI-ALBI scores ranged from 0 to 3. The patients with a score of 0 were considered to have a low PNI-ALBI grade (PNI-ALBI grade 0), and the patients with scores of more than 0 were defined as high PNI-ALBI grade (PNI-ALBI grade 1). Among all the patients in this study, 68 (61.8%) patients were reclassified into PNI-ALBI grade 1, and 42 (38.2%) were into PNI-ALBI grade 0. Spearman correlation was performed to find that PNI was highly correlated with PNI-ALBI grade (*r* = 0.814, *P* < 0.01).

### Analyses for OS

**Figure 3:**
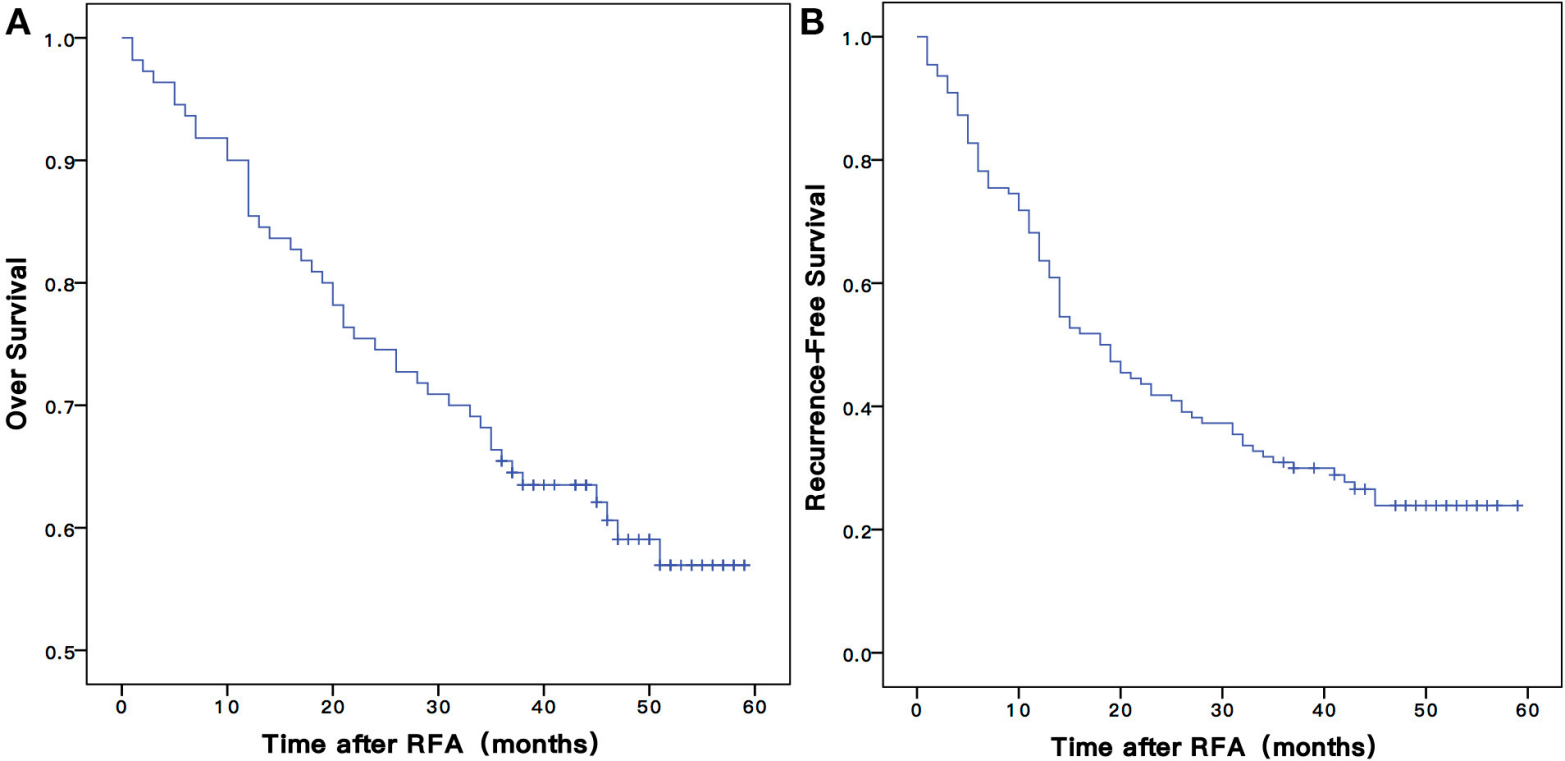
The Kaplan-Meier overall **(A)** and recurrence-free **(B)** survival curves for early stage HCC patients undergoing RFA of this study. RFA, radiofrequency ablation.

The mean follow-up time was 47.5 ±14.8 months, and in between, 45 (40.9%) patients died. The 1-, 3-, and 5-years OS rates of patients were 80.0, 30.9, and 23.9%, respectively (Figure 3A).

**Figure 4:**
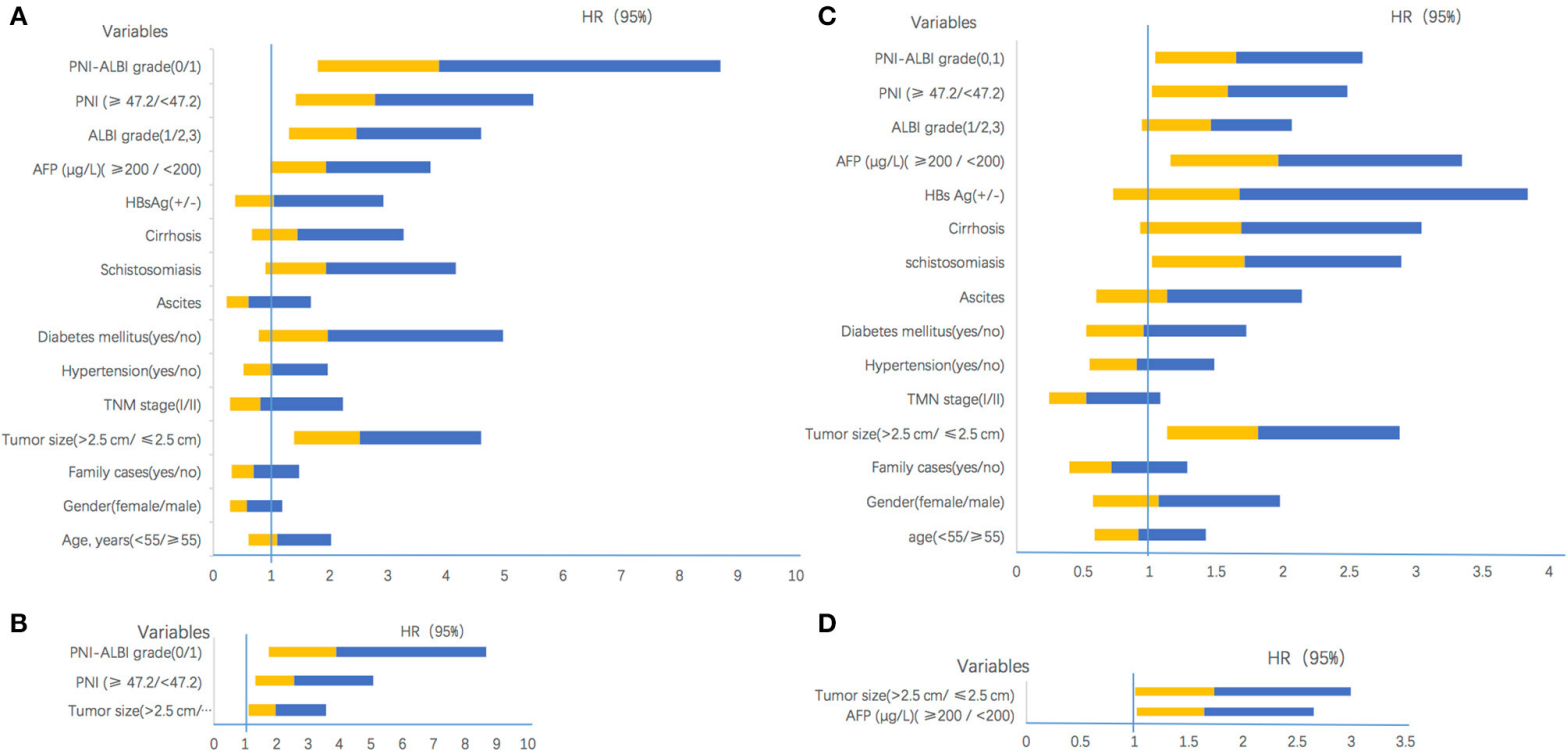
Confidence interval charts of univariate and multivariate analyses. **(A)** The univariate analyses results for overall survival. **(B)** The multivariate analyses results for overall survival. **(C)** The univariate analyses results for recurrence-free survival. **(D)** The multivariate analyses results for recurrence-free survival. HR, hazard ratio; CI, confidence interval; TMN, tumor-node-metastasis; HBs Ag, hepatitis B surface antigen; AFP, α-fetoprotein; PNI, prognostic nutritional index; ALBI, albumin-bilirubin.

As shown in Table 2, the univariate analysis showed potential association of OS with the following parameters: tumor size [HR = 2.524, 95% confidence interval (CI) = 1.387–4.593, *P* = 0.003], schistosomiasis (HR = 1.937, 95% CI = 0.902–4.161, *P* = 0. 0.040), high AFP level (HR = 1.924, 95% CI = 0.993–3.729, *P* = 0.050), ALBI grade (HR = 2.447, 95% CI = 1.301–4.604, *P* = 0.006), PNI grade (HR = 2.778, 95% CI = 1.406–5.488, *P* = 0.003), and PNI-ALBI grade (HR = 3.876, 95% CI = 1.792–8.690, *P* = 0.001). The result is shown in the CI chart (Figure 4A).

**Table 2:** Univariate and multivariate analyses of baseline prognosticators for overall survival in patients with early-stage hepatocellular carcinoma after radiofrequency ablation.

**Variables**	**Univariate analysis**		**Multivariate analysis**	
	**HR (95% CI)**	***P*-value**	**HR (95% CI)**	***P*-value**
Age, years (<55/≥55)	1.097 (0.594–2.029)	0.766		
Gender (female/male)	0.588 (0.290–1.191)	0.140		
Family cases (yes/no)	0.679 (0.316–1.462)	0.323		
Tumor size (>2.5/ ≤ 2.5 cm)	2.524 (1.387–4.593)	0.003	1.966 (1.091–3.545)	0.025
TNM stage (I/II)	0.796 (0.285–2.225)	0.663		
Hypertension (yes/no)	0.995 (0.503–1.970)	0.989		
Diabetes mellitus (yes/no)	1.959 (0.773–4.968)	0.157		
Ascites	0.598 (0.214–1.672)	0.327		
Schistosomiasis	1.937 (0.902–4.161)	0.040		
Cirrhosis	1.456(0.650–3.261)	0.361		
HBsAg (+/–)	1.041 (0.373–2.910)	0.938		
AFP (μg/L) (≥200/ <200)	1.924 (0.993–3.729)	0.050		
ALBI grade (1/2,3)	2.447 (1.301–4.604)	0.006		
PNI (≥ 47.2/ <47.2)	2.778 (1.406–5.488)	0.003	2.558 (1.289–5.078)	0.007
PNI-ALBI grade (0/1)	3.876 (1.792–8.690)	0.001	3.876 (1.729–8.690)	0.001

*HR, hazard ratio; CI, confidence interval; TNM, tumor-node-metastasis; HBsAg, hepatitis B surface antigen; AFP, α-fetoprotein; PNI, prognostic nutritional index; ALBI, albumin-bilirubin*.

However, in the multivariate analysis, only tumor size (HR = 1.966, 95% CI = 1.091–3.545, *P* = 0.025), PNI (HR = 2.558, 95% CI = 1.289–5.078, *P* = 0.007), and PNI-ALBI grade (HR = 3.876, 95% CI = 1.729–8.690, *P* = 0.001) were independent risk factors for OS (Figure 4B). It confirmed that the PNI-ALBI grade was a strong predictor of OS. The predictive abilities for OS of PNI-ALBI, ALBI, and PNI were compared.

**Figure 5:**
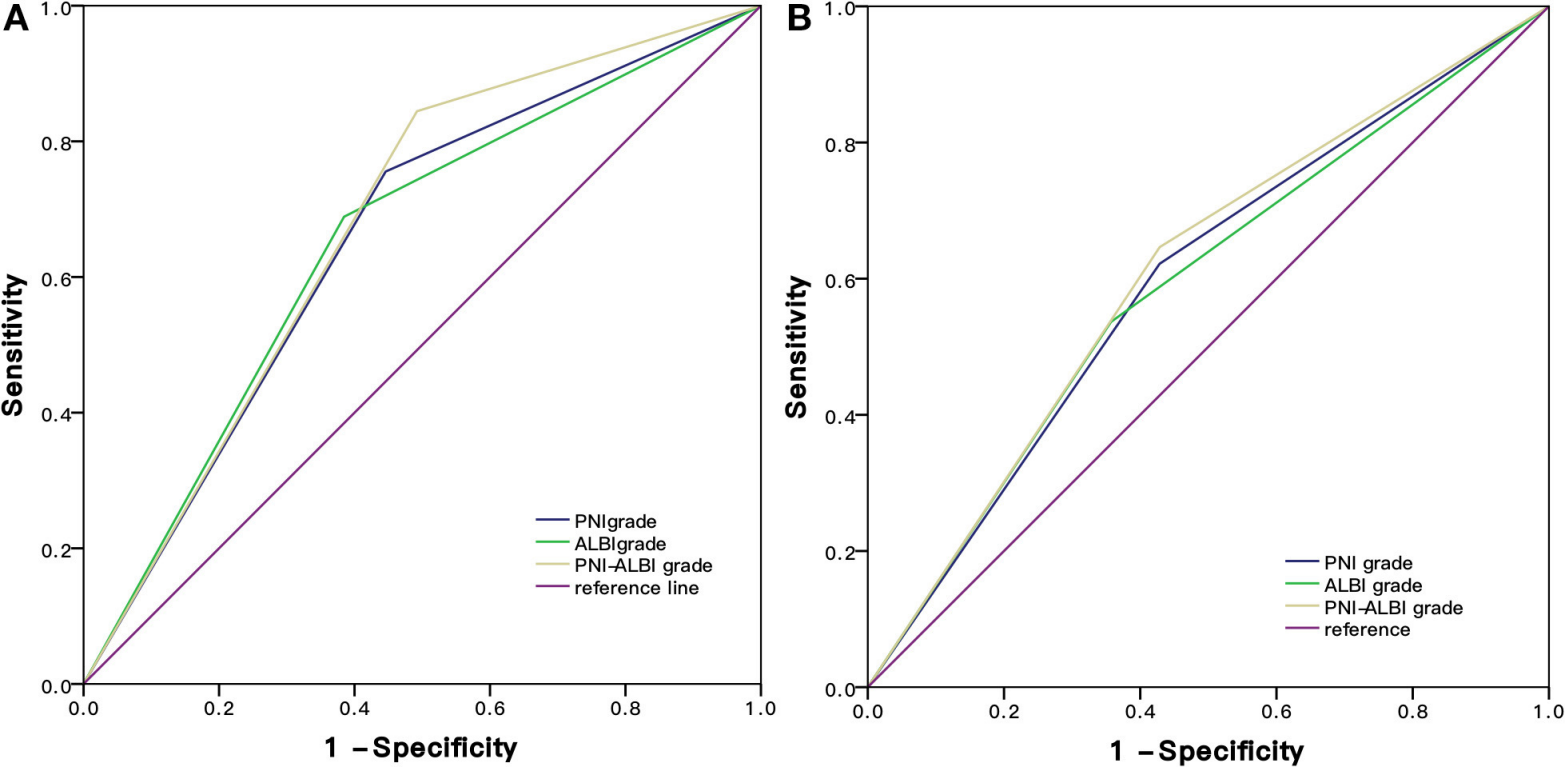
Comparison of the AUC of PNI-ALBI grade, PNI, and ALBI in predicting survival **(A)** and postoperative recurrence **(B)**. RFA, radiofrequency ablation; AUC, area under the receiver operating characteristic curve; PNI, prognostic nutritional index; ALBI, albumin-bilirubin.

Based on the ROC curves, the AUC of PNI-ALBI grade was 0.676, PNI grade was 0.655, and ALBI grade was 0.652. The PNI-ALBI had the highest AUC, which indicated that the PNI-ALBI might be a better factor to predict the OS of the patients with HCC after RFA (Figure 5A).

### Analyses for RFS

There were 82 (74.5%) patients who suffered from recurrence during the follow-up periods. The 1-, 3-, and 5-years RFS rates were 63.6, 53.2, and 35.0%, respectively (Figure 3B). The univariate Cox proportional hazards model showed that tumor size (HR = 1.808, 95% CI = 1.135–2.882, *P* = 0.013), schistosomiasis (HR = 1. 713, 95% CI = 1.014–2.895, *P* = 0.044), AFP (HR = 1.962, 95% CI = 1.152–3.342, *P* = 0.013), and PNI-ALBI grade (HR = 1.647, 95% CI = 1.043–2.600, *P* = 0.032) were independent risk factors for RFS, as shown in Table 3. But in multivariate Cox proportional hazards model, only the AFP (HR = 1.732, 95% CI = 1.003–2.991, *P* = 0.049) and the size of tumor (HR = 1.640, 95% CI = 1.015–2.647, *P* = 0.43) turned out to be independent predictors for better RFS (Figures 4C,D).

**Table 3:** Univariate and multivariate analyses of baseline prognosticators for recurrence-free survival in patients with early-stage HCC after RFA.

**Variables**	**Univariate analysis**		**Multivariate analysis**	
	**HR (95% CI)**	***P*-value**	**HR (95% CI)**	***P*-value**
Age (<55/≥55)	0.908 (0.581–1.418)	0.671		
Gender (female/male)	1.068 (0.578–1.974)	0.833		
Family cases (yes/no)	0.708 (0.390–1.283)	0.254		
Tumor size (>2.5/ ≤ 2.5 cm)	1.808 (1.135–2.882)	0.013	1.640 (1.015–2.647)	0.43
TNM stage (I/II)	0.517 (0.249–1.075)	0.077		
Hypertension (yes/no)	0.900 (0.543–1.490)	0.681		
Diabetes mellitus (yes/no)	0.951 (0.526–1.721)	0.868		
Ascites	1.135 (0.601–2.145)	0.696		
Schistosomiasis	1.713 (1.014–2.895)	0.044		
Cirrhosis	1.682 (0.929–3.046)	0.086		
HBsAg (+/–)	1.668 (0.726–3.833)	0.228		
AFP (μg/L) (≥200/ <200)	1.962 (1.152–3.342)	0.013	1.732 (1.003–2.991)	0.049
ALBI grade (1/2,3)	1.457 (0.942–2.066)	0.091		
PNI (≥47.2/ <47.2)	1.587 (1. 012–2.488)	0.044		
PNI-ALBI grade (0, 1)	1.647 (1.043–2.600)	0.032		

*HCC, hepatocellular carcinoma; RFA, radiofrequency ablation; HR, hazard ratio; CI, confidence interval; TNM, tumornodemetastasis; HBsAg, hepatitis B surface antigen; AFP, a-fetoprotein; PNI, prognostic nutritional index; ALBI, albuminbilirubin*.

Based on the ROC curves, the AUC of PNI-ALBI grade was 0.609, PNI was 0.590, and ALBI grade was 0.597 (Figure 5B). For predicting postoperative RFS, the PNI-ALBI grade had the highest AUC, followed by ALBI grade and PNI.

### The Difference Between the Patients With Low and High PNI-ALBI Grade

**Figure 6:**
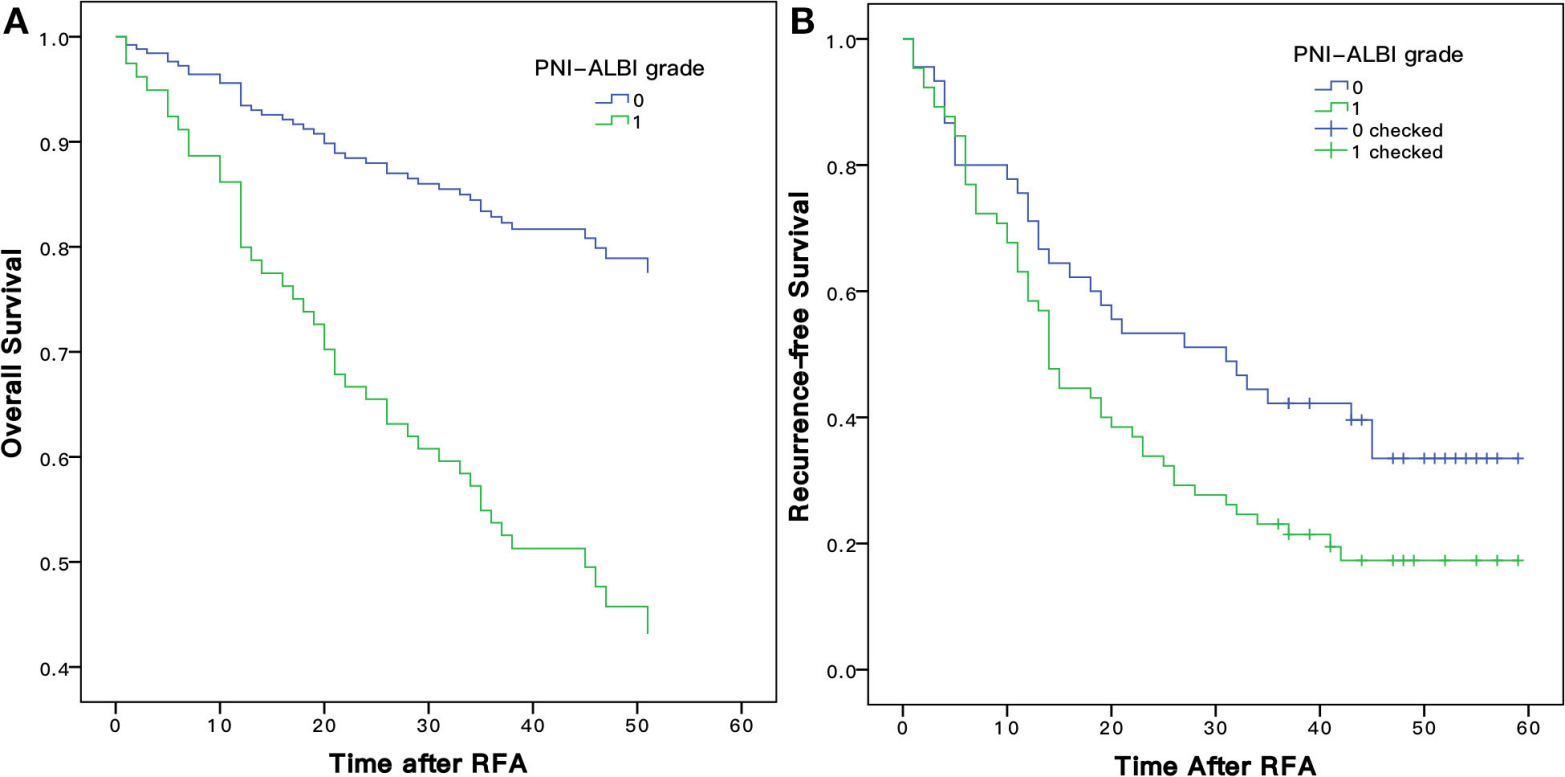
Comparison of Kaplan-Meier overall **(A)** and recurrence-free **(B)** survival curves for early stage HCC patients with different PNI-ALBI grade. PNI, prognostic nutritional index; ALBI, albumin-bilirubin; HCC, hepatocellular carcinoma.

As shown in Figure 6A, the 1-, 3-, and 5-years OS rates were 93.3, 82.8, and 79.9%, respectively, for patients with low PNI-ALBI grade. For patients with high PNI-ALBI grade, they were 71.1, 39.6, and 33.5%, respectively, and the difference was statistically significant (*P* < 0.001). As shown in Figure 6B, the 1-, 3-, and 5-years RFS rates for patients with low PNI-ALBI grade were 80.0, 52.2, and 41.4%, respectively; and 58.8, 23.1, and 17.3%, respectively, for patients with high PNI-ALBI grade (*P* < 0.001).

We compared the clinicopathological characteristics of patients with different PNI-ALBI grades (Table 4). Patients with high PNI-ALBI grade had a higher incidence of larger tumor size (≥2.5 cm), ascites, splenomegaly, cirrhosis, AFP, lower albumin, and higher total bilirubin compared with low PNI-ALBI grade.

**Table 4:** Comparison of clinicopathologic characteristics between patients with low PNI-ALBI grade and high PNI-ALBI grade.

**Clinicopathological**	**Low PNI-ALBI**	**High PNI-ALBI**	***P*-value**
**feature**	**grade**	**grade**	
**Age**			
<55 years	18 (42.9%)	25 (36.8%)	0.525
≥55 years	24 (57.1%)	43 (63.2%)	
**Gender**			
Female	5 (11.9%)	13 (19.1%)	0.320
Male	37 (88.1%)	55 (80.9%)	
**Family cases**			
Yes	7 (16.7%)	9 (13.2%)	0.828
No	35 (83.3%)	59 (86.8%)	
**Tumor size**			
>2.5 cm	6 (14.3%)	24 (35.3%)	0.016
≤ 2.5 cm	36 (85.7%)	44 (64.7%)	
**TNM stage**			
I	39 (92.9%)	63 (92.6%)	0.97
II	3 (7.1%)	5 (7.4%)	
**Hypertension**			
Yes	10 (23.8%)	17 (25.0%)	0.538
No	32 (76.2%)	51 (75.0%)	
**Diabetes mellitus**			
Yes	9 (21.4%)	10 (14.7%)	0.439
No	33 (78.6%)	58 (85.3%)	
**Ascites**			
Yes	2 (4.8%)	12 (17.6%)	0.042
No	40 (95.2%)	56 (82.4%)	
**Splenomegaly**			
Yes	19 (45.2%)	60 (88.2%)	<0.001
No	23 (54.8%)	8 (11.8%)	
**Cirrhosis**			
Yes	25 (59.5%)	62 (91.2%)	<0.001
No	27 (64.3%)	6 (8.8%)	
**HBsAg**			
Present	37 (88.1%)	63 (92.6%)	0.332
Absent	6 (14.3%)	5 (7.4%)	
**AFP (μg/L)**			
Normal	39 (92.9%)	51 (75.0%)	0.022
Abnormal	3 (7.1%)	17 (25.0%)	
WBC (×10^3^/μL)	5.09 ± 1.49	3.96 ± 1.73	0.382
Platelet count (×10^9^/L)	136.90 ± 61.03	87.41 ± 46.29	0.157
Prothrombin time (s)	12.10 ± 1.74	18.44 ± 44.16	0.146
Albumin (g/L)	43.74 ± 2.65	36.18 ± 4.11	0.002
Total bilirubin (μmol/L)	15.57 ± 7.62	21.12 ± 9.93	0.028

*HR, hazard ratio; CI, confidence interval; TNM, tumor–node–metastasis; HBsAg, hepatitis B surface antigen; AFP, α-fetoprotein; PNI, prognostic nutritional index; ALBI, albumin–bilirubin*.

## Discussion

HCC ranks third in the cancer-related causes of the deaths worldwide (22). Ablation is the recommended treatment for early HCC, and RFA is the most mature and widely used treatment among them (23). It is recommended as the first-line therapy for HCC <3 cm in diameter, as it has a similar survival outcome compared with surgical resection (24). Because of the metabolic function of the liver, the PNI, which was initially designed to evaluate the immunological and nutritional status of patients after surgery of the gastrointestinal tract, was applied in patients with HCC and turned out to be an independent predictor of OS (15, 25–27). Patients with a lower PNI tend to have a worse OS. Low PNI may be caused by hypoalbuminemia and/or lymphocytopenia. The question arises: How do hypoalbuminemia and lymphocytopenia contribute to tumor development and progression? Lymphocytes are important in the adaptive immune system that acts against cancer. They are the cellular foundation for cancer immunosurveillance and immunoediting, and a previous study had proven that a higher lymphocyte count could guarantee an effective antitumor cellular immune response. Likewise, a lower lymphocyte count might weaken the antitumor immune defense, indicating a poor prognosis (28). Hypoalbuminemia in patients with HCC is caused by an impaired liver function due to the underlying chronic liver disease and is also associated with a sustained systemic inflammatory response, either as a host reaction or from the tumor itself.

Rather than advanced-stage diseases, PNI was a better prognostic predictor in patients with an early stage (29), and all the patients in our study were in the early stage. A previous study found that ALBI grade, known as a new assessment method for the hepatic function, was proposed, which was reported to be better than CP and Model for End-stage Liver Disease (MELD) scale in the assessment ability for hepatic function (30). The MELD score was calculated by three objective variables, which were total bilirubin, creatinine, and international normalized ratio (INR). But INR was reported not to sufficiently reflect coagulopathy and consequently liver function in liver cirrhosis (31). The CP score was inferior in terms of stability, because two parameters in the score, namely, ascites and encephalopathy, were variable and were mostly subjective to the observer. In comparison, ALBI grade is a simple and more objective way to assess liver function and therefore could be a better tool in the evaluation of patients with HCC (32). HCC patients with relatively well-preserved hepatic function are more likely to receive appropriate therapy, and poor liver function is associated with increased treatment-related toxicity and inferior survival. Also, patients with better liver function can recover faster with fewer complications (33). More and more studies confirmed that ALBI grade is an independent predictor of OS in patients with HCC (34, 35).

It is hypothesized that the combination of PNI and ALBI would increase the accuracy of prognostic evaluation after RFA for HCC, as the combination could evaluate both the nutritional status and the hepatic function of patients with HCC. In this retrospective study, 110 patients with newly diagnosed HCC were included, and all the patients received the RFA. The multivariate analysis and univariate analysis results showed that the PNI-ALBI grade was an independent influencing factor for the OS of the patients with HCC after RFA. More importantly, the performance of PNI-ALBI grade in predicting OS of the HCC patients was better than PNI or ALBI alone. Patients with high PNI-ALBI grades were more likely to have a worse OS. But for RFS, the univariate analysis found that the AFP and PNI-ALBI grade seems to be a predictor; however, in multivariate analysis, the prognostic values of the ALBI and PNI for RFS are uncertain in patients with early-stage HCC receiving RFA as initial therapy. Okamura et al. found the PNI predicts only OS and not RFS in HCC patients after hepatectomy (36). Chu et al. also demonstrated that an elevated AFP level of ≥200 ng/mL was a significant factor associated with RFS by univariate and multivariate analyses, whereas PNI had limited prognostic value for RFS in early-stage HCC patients undergoing RFA (15). However, a study conducted by Chan et al. showed that the PNI could be an important prognostic parameter for HCC patients who underwent hepatectomy (37). Regarding ALBI, several studies have evidenced that it could have a predictive value in OS. But fewer studies were performed to evaluate the predictive role of ALBI in estimating HCC recurrence (38). No clear explanation for this difference is available at present. Further research with larger sample size and longer follow-up time on this issue may show the PNI and ALBI to be a significant predictor of both OS and RFS. A significant difference in the PNI-ALBI grade was identified in the current univariate analysis for RFS.

Our results indicated that the PNI-ALBI grade could better reflect the long-term survival for patients with HCC after RFA, compared with either score alone. The results of our study can help us to identify patients who had a potentially poor prognosis and take interventions before the RFA.

Although, to our best knowledge, this is the first study to combine PNI and ALBI to evaluate the prognostic value in patients with early-stage HCC after RFA, this study had several potential limitations. First, this was a retrospective study. Second, this was a single-center study, and patients involved were confined to the east of China. Third, the sample size of the study was limited, and a further larger sample size cohort is needed. Fourth, some other well-known indicators, including the platelet-to-lymphocyte ratio, C-reactive protein level, and Glasgow prognostic score, were not evaluated in our cohort, although they have been proposed earlier as prognostic factors for patients with HCC.

In conclusion, this study demonstrated that the PNI-ALBI grade was a more accurate marker for predicting the OS of patients with early-stage HCC treated with RFA in comparison to PNI or ALBI alone. Patients with high PNI-ALBI grade are more likely to have a worse outcome, which suggests that we should check out the patients' liver function and nutritional status before performing the RFA.

## Data Availability Statement

All datasets generated for this study are included in the article/Supplementary Material.

## Ethics Statement

The studies involving human participants were reviewed and approved by the Research Ethics Committee of The First Affiliated Hospital of Zhejiang University. The ethics committee waived the requirement of written informed consent for participation.

## Author Contributions

JP conceived the study and wrote the manuscript. SC collected the data. JP and GT performed statistical analyses. TJ provided support and helped with manuscript revision. All authors were involved in analyzing the results.

## Conflict of Interest

The authors declare that the research was conducted in the absence of any commercial or financial relationships that could be construed as a potential conflict of interest.
